# CD1a-positive infiltrating-dendritic cell density and 5-year survival from human breast cancer

**DOI:** 10.1038/sj.bjc.6601114

**Published:** 2003-07-29

**Authors:** B J Coventry, J Morton

**Affiliations:** 1 Breast, Endocrine and Surgical Oncology Unit and Tumour Immunology Laboratory, Department of Surgery, University of Adelaide, Royal Adelaide Hospital, North Terrace, Adelaide, South Australia 5000, Australia

**Keywords:** CD1a, dendritic cells, breast cancer, survival

## Abstract

Infiltrating CD1a^+^ dendritic cells (DCs) have been associated with increased survival in a number of human cancers. This study investigated DC infiltration within breast cancers and the association with survival. Classical established prognostic factors, of tumour size, lymph node status, histological grade, lympho-vascular invasion, the KI-67 (MIB-1) fraction and the Nottingham Prognostic Index (NPI) were also compared. A total of 48 breast cancer patients were followed from the time of surgery and CD1a density analysis for 5 years or until death. Our data set validated previous studies, which show a relationship between survival and the NPI (*P*<0.001), tumour size (*P*<0.01) and lymph node status (*P*<0.05). Although more patients were alive at the 5-year time point in the group with higher CD1a DC density than the lower CD1a DC group, this failed to reach statistical significance at the *P*=0.05 level. Analysis at 10 years postsurgery is required to investigate the association further.

Breast cancer is the most common terminal cancer in women in developed Western societies (Australian Bureau of Statistics, 2002). The overall survival rate at 5 years is approximately 70%; however, prognostic factors can define subgroups within the larger group that may refine this figure to as high as 90% or to less than 20% in individual cases where certain prognostic factors are known (Pereira *et al*, 1995; Veronesi *et al*, 1995). The primary tumour pathology can predict the extent of local or distant tumour spread and augment clinical investigational information to better select appropriate surgery, adjuvant chemotherapy and radiotherapy and to assist the understanding of likely clinical outcome. Lymph node involvement, tumour size and the histological grade of the tumour (Dalton *et al*, 1994; Page *et al*, 1998), in order of prognostic strength of association, are able to predict metastasis, recurrence and survival (Nixon *et al*, 1996). These have been integrated together to form the Nottingham Prognostic Index (NPI) (Galea *et al*, 1992). The validity of the NPI has been shown repeatedly in breast cancer patients over 20 years such that patients with an NPI of <3.4 have been found to have an overall 15-year survival rate of 80%, those with an NPI of 3.4–5.4 have a moderate survival rate of 42%, while patients whose NPI is >5.4 have a 15-year survival rate of only 13%. Another feature that has been associated with clinical outcome is Ki-67 expression (expressed by all cells not in G_0_ phase), or the use of the MIB-1 antibody that reacts with part of the Ki-67 antigen (Pinder *et al*, 1995). Prognostic indices, such as the Nottingham Index, are important, useful tools that focus on tumour characteristics, but increasing interest is being directed towards the immunological responses that occur within the tumour microenvironment (Austyn, 1993; Coventry, 1999). Studies have shown that activated lymphocytes can cause tumour-specific cell lysis and that local immunosuppression appears to be present within the tumour microenvironment rendering the antitumour immune response ineffective (Whiteside *et al*, 1986a, 1986b; Vitolo *et al*, 1992, 1993; Coventry *et al*, 1996, 2002). A possible reason for the poor antitumour immune response may be the reduced number and activation status of dendritic cells (DCs) in human breast cancers. We have previously shown that DCs are absent, or only present in very low numbers, in most human breast cancers using CD1a and other markers and that DCs are poorly activated (Coventry *et al*, 1997; Hillenbrand *et al*, 1999; Coventry *et al*, 2002).

CD1a has been reviewed in detail elsewhere (Coventry, 1999); however in brief, CD1a is a glycosylated type I transmembrane polypeptide chain noncovalently associated with *β*2 microglobulin (Shaw, 1998; Sieling, 1998). There is significant sequence homology between CD1a, and class I and II molecules of the major histocompatibility complex (MHC) (Shaw, 1998). The peptide binding site, while similar to MHC molecules, is longer, deeper and more hydrophobic (Zeng *et al*, 1997). CD1a has been used as a marker for DCs in a range of human tumours and the density of CD1a DC has been associated with clinical outcome. Dendritic Cell density, using a variety of markers including CD1a, has been reported to correlate with survival in a range of human tumour types including lung (Furukawa *et al*, 1985; Zeid and Muller, 1993), colon (Ambe *et al*, 1989), gastric (Tsujitani *et al*, 1987), nasopharyngeal (Gallo *et al*, 1991a; laryngeal (Gallo *et al*, 1991b)) and tongue carcinomas (Goldman *et al*, 1998).

A recent study demonstrated no apparent significant relationship between DC density and survival, using S-100 as a marker for DC, in formalin-fixed paraffin-embedded breast cancer tissue samples (Lespagnard *et al*, 1999).

The aim of this study was to assess the relationship between CD1a DC density in fresh human breast cancer tissues, with disease-specific 5-year postsurgery survival. The possible association of CD1a density and other established prognostic markers was also investigated.

## METHODS

### Patient sample selection

Fresh tissue was taken from 51 female patients following surgical resection for infiltrating ductal breast carcinoma between 1990 and 1994 at the Royal Adelaide Hospital. Three patients were excluded due to unavailability of 5-year follow-up data, giving a sample size of 48. Of these breast carcinomas, 42 tumours were infiltrating ductal in type and six were ductal carcinomas *in situ* (DCIS). Ethical approval was given for the studies by the RAH Human Ethics Committee.

### Tissue processing

Tissue was embedded in OCT (Tissue Tek, Miles Scientific, Illinois) and snap-frozen in liquid nitrogen. Serial sections (4 *μ*m) were cut using a cryostat (Leica, CM1500, Germany) onto gelatinised slides, acetone fixed (10 min) and air-dried.

### Monoclonal antibodies and immunohistochemical techniques

Mouse anti-human monoclonal antibodies (Mab), diluted with 10% normal horse serum (NHS, Sigma-Aldrich, Sydney) in phosphate-buffered saline (PBS), were varied in concentration to determine the optimum dilutions: CD1a at 1 : 1000 (Dako, Sydney), Cam 5.2 at 1 : 500 (Becton Dickinson, Sydney, Australia), murine isotype controls IgG1, IgG2a (supernatants at 1 : 16, L Ashman, IMVS Adelaide, Australia), second antibody biotinylated rabbit anti-mouse IgG/IgM at 1 : 500 (Dako, Sydney) and streptavidin–HRP at 1 : 1000 (Pierce, USA).

All reactions were carried out with PBS washes in triplicate between incubations at room temperature. Sections were blocked with 10% NHS (30 min), incubated overnight in a humidified chamber with primary antibody, followed by biotinylated second antibody incubation (1 h). Endogenous peroxidase blockade (0.5% hydrogen peroxide; methanol, 20 min) was followed by streptavidin–HRP incubation (1 h). High-sensitivity nickel chloride-enhanced diaminobenzidene (nickel DAB) techniques were used and sections were counterstained with methyl green (Coventry *et al*, 1994; 1995; Hillenbrand *et al*, 1999).

### Visual quantitation

Cell density was quantified as the mean of 50 random high power fields (hpf) per frozen section (field size 0.375 mm diameter, 0.11 mm^2^, × 400 magnification) using relevant stains for respective cell types to enable a comparison of the density and distribution of different cell types (Dunnill, 1968).

### Identification of DC

Anti-CD1a Mab was used to identify DC in tissue sections. Assessment of DC morphology was also performed and assisted identification.

### Identification of tumour cells

The anti-cytokeratin Mab, Cam 5.2, was used to identify tumour (and normal) epithelial cells in the adenocarcinomas, to exclude the possibility of CD1a staining of epithelial cells as opposed to DC (Makin *et al*, 1984; Hillenbrand *et al*, 1999).

### Follow-up data collection

The 5-year follow-up data were obtained from hospital case notes and clinical records, general practitioners, surgeons and the government registry of Deaths. This included interstate follow-up. The patient details included age at diagnosis/surgery, date of diagnosis/surgery, duration of follow-up, date of death, cause of death, date of metastasis/local recurrence and the therapy received.

### Pathology data collection

The original pathology reports were obtained for all 48 patients. These were used to determine the tumour microscopic size, grade, lymph node status, presence of lympho-vascular invasion and the Ki-67 (or MIB-1) fraction. The NPI was calculated using this data. The tumour grade was assessed according to the Scarfe, Bloom and Richardson system identifying grade I, II or III for each invasive tumour (Bloom and Richardson, 1957; Scarff and Tolono, 1968). Ductal carcinoma *in situ* was also noted separately to any invasive component, and specifically tabulated if this was the only tumour present.

### Causes of death

For the patients who died, the cause of death was established through the South Australian Department of Births, Deaths and Marriages. Breast cancer-specific mortality was used for the mortality calculations.

### Statistics

All statistics were performed with the assistance of statisticians (see Acknowledgments) using the SAS^©^ program. The primary outcome measure was breast cancer-specific survival at 5 years from the time of diagnosis. Statistical correlations and associations between survival and the prognostic factors (CD1a density, tumour size, lymph node status, histological grade, the NPI and the Ki-67 (or MIB-1) fraction) were performed. A correlation between CD1a density and the NPI was investigated using Fisher's Exact *χ*^2^ test due to the small sample size used in the study. The prognostic indicators were treated as categorical, instead of continuous variables, due also to the small sample size. Indices were used in standard methodological groupings: the NPI, as recommended by Galea *et al* (1992); lymph node metastases into present or absent; and histological grade was classified as Bloom and Richardson grades I, II and III or separately as DCIS. CD1a density, tumour size and Ki-67 fraction were divided into two groups, either side of the respective medians. The groups were therefore defined around the medians of: (i) CD1a density 0.78 (cells/hpf), (ii) tumour size 25 (mm) or (iii) Ki-67 fraction 45 (%), respectively. Medians were chosen on statistical grounds to limit influence from any non-normal distribution of data. For CD1a density, several cutoff levels lower and higher than the median were also chosen for extended analysis.

## RESULTS

The data obtained are shown in Table 1

**Table 1:** Patient data for the study group

	**Median**	**Range**
*n*=48		
Age at diagnosis (years)	60.5	26–84
Age (survivors at 5 years)	67	31–89
Age (died before 5 years)	60.5	41–87
CD1a count (cells/hpf)	0.78	0–14.85
Tumour size (mm)	25	8–200
Nodes removed total	12	1–24
NPI	3.55	1.02–7
		
**Number in category**		
Tumour grade		
DCIS	6	
I	8	
II	24	
III	10	
DCIS component present	38	
Positive nodes	14	
Vascular invasion	12	
Tamoxifen	21	
Radiotherapy	22	
Chemotherapy	15	
Family history	7	
Metastases at diagnosis	2	
Metastases at 5 years	10	
Ki-67 (+ve >20%)	15	
ER (+ve >50%)	24	
PR (+ve >50%)	20	

. The median age was 60.5 years at diagnosis with a range from 26 to 84 years. Survival data were obtained for 48 patients with CD1a density (and other) measurements. Of these, 12 women had died before the 5-year time point from surgery/diagnosis. All of these deaths were attributable to breast cancer.

### Traditional tumour-associated prognostic factors and survival

Tumour size <25 mm diameter was demonstrated to be a prognostic index for survival (*P*<0.02). The absence of lymph node metastasis was also found to be associated with survival (*P*<0.05). Limited data on nine pathology reports prevented calculation of the NPI, leaving 39 patients where the NPI was available. Values ranged from 1.02–7, with 13 women in the good prognostic group, 20 in the moderate group and six women in the poor prognostic group. The NPI was strongly associated with survival (*P*<0.001), with 100% of women dying in the poor prognostic group.

The distribution of tumours according to grade was: six DCIS; eight grade I; 24 grade II and 10 grade III. The histological grade of the tumour was not significantly associated with survival with a *P*-value of 0.08. The Ki-67 (MIB-1) fraction was also not found to be associated with survival (*P*=0.091) in this series. The presence of lympho-vascular invasion within the tumour was associated with mortality (*P*=0.0126). As expected, the presence of systemic metastases was associated with reduced survival (*P*<0.01). However, in contrast, the presence of local–regional tumour recurrence was not associated with increased risk of death (*P*=0.304).

### CD1a DC density and survival

**Figure 1:**
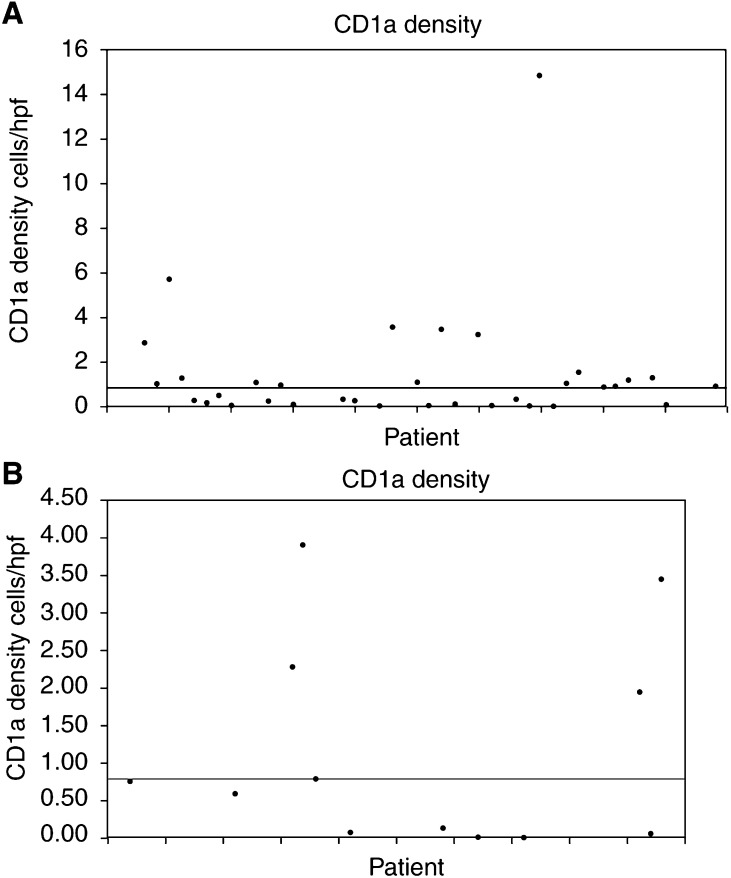
Scattergram showing the dispersion of CD1a density data around the median (A) for patients alive at 5 years postsurgery and (**B**) for patients dead at 5 years postsurgery.

CD1a staining was confined to DC with no staining of Cam 5.2-positive epithelial cells in serial sections. The CD1a count was available for all 48 patients ranging from 0 to 14.85, with a median of 0.78 cells per high power field. In 40 out of 48 breast cancers, CD1a-positive cells were detectable using these methods. Comparison of raw data is shown by the dispersion of CD1a density data around the median for patients alive (Figure 1A

) and dead (Figure 1B) at 5 years postsurgery. A higher death rate (32%) was observed in the patients with a lower CD1a count, compared to 18% mortality for the group with a higher CD1a density within their breast tumours. However, the CD1a count was not significantly associated with 5-year survival (*P*=0.331) (Table 2

**Table 2:** CD1a density and survival

**(*n*=47)**	**Dead (%)**	**Alive (%)**
Low CD1a	32	68
High CD1a	18	82

**Figure 2:**
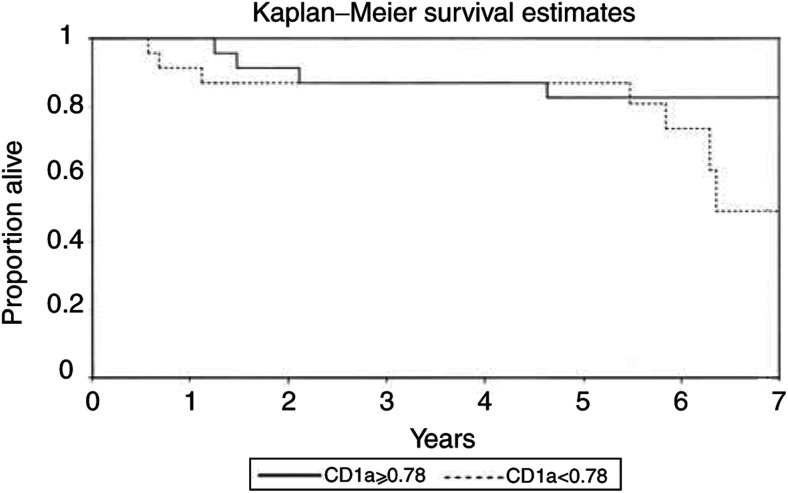
Survival curves of patients with CD1a densities either less than the median of 0.78 cells/hpf or 0.78 and greater using the Kaplan–Meier method.

Note that 32% of the patients with low CD1a density died within 5 years, while only 18% of those with a count died (*n*=47). The association between the number of CD1a^+^ DCs was not significantly associated with survival at 5 years postsurgery (P=0.331).

). The data were also analysed using the Kaplan–Meier method relating the survival curves of patients with CD1a densities either less than the median of 0.78 cells/hpf or 0.78 and greater (Figure 2

).

The association between CD1a count and the NPI was also not significant (*P*=0.486), but again the raw data showed an apparent trend towards a higher, or a worsening, NPI and lower CD1a density. The sample (*n*=39) for this statistic was necessarily small due to a lack of complete data for the calculation of the NPI (Table 3

**Table 3:** Cd1a density and NPI

**(*n*=39)**	**Low CD1a (%)**	**High CD1a (%)**
NPI>5.4 (poor prognosis)	80	20
NPI 3.4–5.4 (moderate prognosis)	50	50
NPI<3.4 (good prognosis)	46	54

The association between CD1a and the NPI shows a nonsignificant (*P*=0.486) trend. Note that 20% of the patients with a higher CD1a density had a high (unfavourable) NPI, while 80% of the patients with a low CD1a density had an unfavourable NPI. The trend was reversed for a low (more favourable) NPI.

).

Analyses were performed between CD1a and tumour size, grade, nodal status, Ki-67 (MIB-1), presence/absence of metastases, presence/absence of recurrence, presence/absence of lympho-vascular invasion. No statistical association at the *P*=0.05 level could be found between CD1a density and any of these prognostic variables.

## DISCUSSION

Dendritic cell density in human tumour tissues has been investigated using a variety of markers and methods. Principally, CD1a and S-100 have been used for tissue section investigations, but more recently, CD83, CD86, CMRF-44/-56 and other markers have been utilised. CD1a molecules have been reviewed elsewhere (Coventry, 1999), but appear to have a role in antigen presentation showing relatively tightly restricted expression on DC. A very small subgroup of monocytes in adults and thymocytes in early life can also express CD1a. CD1a-positive DCs have been reported to be present within breast cancers from very early, preinvasive DCIS lesions to invasive ductal carcinomas, but there was no statistical correlation between DC density and the grade of the tumour (Hillenbrand, 1999). On the other hand, S-100-positive DC do appear to show some correlation with the breast tumour grade (Bell *et al*, 1999; Lespagnard *et al*, 1999), indicating that CD1a and S-100 define somewhat different populations of DCs in breast cancers. S-100 is a small acidic regulatory protein involved in a wide range of cellular processes and exhibits relative tissue specificity for DCs and cells of neural origin, including melanoma cells (Heizmann *et al*, 2002). The density of DCs, using either CD1a or S-100 as markers for DC, has been reported for a variety of human cancers including cervix (Younes *et al*, 1968; Bethwaite *et al*, 1996), ovary (Eisenthal *et al*, 2001), lung (Bassett *et al*, 1974; Fox *et al*, 1989; Zeid and Muller, 1993), larynx (Schenk, 1980), salivary glands (David and Buchner, 1980), skin (Gatter *et al*, 1980), breast (Hillenbrand *et al*, 1999; Coventry *et al*, 2002), thymus (Hammar *et al*, 1986), oesophagus (Matsuda *et al*, 1990), stomach (Tsujitani *et al*, 1987), pancreas (Dallal *et al*, 2002), thyroid (Schroder *et al*, 1988; Willgeroth *et al*, 1992), colon (Ambe *et al*, 1989), nasopharynx (Nomori *et al*, 1986; Gallo *et al*, 1991a; laryngeal (Gallo *et al*, 1991b)), oral (Kikuchi *et al*, 2002), prostate, bladder and kidney (Bigotti *et al*, 1991; Inoue *et al*, 1993; Troy *et al*, 1998a, 1998b, 1999). Furthermore, DC numbers, as measured using either CD1a or S-100 antibodies, have been positively associated with improved outcome (increased survival) for many of these cancers, although the mechanism remains unclear. S-100-positive DC number has been associated with improved survival for colon, gastric, lung and laryngeal carcinomas (Tsujitani *et al*, 1987; Ambe *et al*, 1989; Gallo *et al*, 1991a, 1991b; Zeid and Muller, 1993). More recently, breast cancer was investigated, but no association of S-100 with survival could be shown (Lespagnard *et al*, 1999).

The data regarding CD1a and survival are more limited. The density of CD1a-positive DC has been directly related to survival for tongue carcinomas (Goldman *et al*, 1998) and reduced tumour recurrence in ovarian carcinoma (Eisenthal *et al*, 2001). Until recently, no study has specifically addressed the relationship of CD1a density in breast cancer to clinical outcome or survival.

Our findings in this study addressed this issue and showed that the density of tumour infiltrating CD1a^+^ dendritic cells did not significantly correlate (at the *P*=0.05 level) with overall survival at the 5-year time point following surgery. Clearly, sample size may have been a possible limitation of our study, since a trend was demonstrated (shown in Table 2). However, the sample size was sufficiently large to demonstrate a statistical correlation between the classical prognostic variables of tumour size, lymph node status, lympho-vascular invasion, metastases and the NPI. Our data lend further support to the findings of Lespagnard *et al* (1999) from a similar study using S-100 as an alternative marker for DC in formalin-fixed archival breast cancer tissues. Notably, their study examined 143 cases and also showed no statistical difference between DC numbers as measured by S-100 expression and 5-year survival. A very recently published study by Iwamoto *et al* (2003) showed similar findings, indicating that CD1a density in breast cancer was not related to clinical outcome or survival. Taken collectively, these findings imply that the density of DC *per se*, as measured by CD1a or S-100, does not appear to act as an independent determinant of overall 5-year survival from breast carcinoma.

These are important findings and indicate that either (i) DC density may not directly determine tumour cell growth, metastasis and outcome in breast cancer or (ii) CD1a and S-100 as conventional measures of DC density may not be sensitive or specific enough for prognostic use.

There is considerable evidence that tumour-specific responses are present in human breast cancers, but that the immune response is inhibited and/or ineffective in most cases. The demonstration of tumour-specific lysis by tumour infiltrating lymphocytes strongly indicates that DCs are important in *in vivo* breast cancer antitumour immunity (Baxevanis *et al*, 1994). The concept of a detectable antitumour response in breast cancer is further supported by other demonstrations of tumour-specific cytolytic and cytokine responses to breast cancer cells utilising different methods (Whiteside *et al*, 1986a, 1986b; Shwartzentruber *et al*, 1992). These findings show that the antitumour immune response in breast cancer is ineffective and that deficient DC function, at least in part, may be responsible for this (Coventry *et al*, 1996; Gabrilovich *et al*, 1997). The maturity and location of DC within breast tumours may not be uniform, such that more mature DCs, expressing differentiation/activation markers such as CD83, have been noted to be located peritumourally around the tumour mass, rather than infiltrating within it (Bell *et al*, 1999; Tsuge *et al*, 2000). Further recent data from two collaborative laboratories, using different methods and samples, demonstrated that the activation of DC was very low or absent within the breast cancer microenvironment using a variety of markers (Coventry *et al*, 2002). The finding that activation was low or absent in tumour-associated DC populations in most breast cancers may have some bearing on the lack of apparent statistical association between CD1a and S-100 expression and survival. The observations that activated, mature DCs were restricted to a peritumoural location rather than within the tumour (Bell *et al*, 1999; Tsuge *et al*, 2000) imply that regional differences around and within breast cancers may be important in determining the capacity of DC to present antigens depending on their location. CD1a^+^ DC may not have the capacity to adequately mature, and present antigen within the tumour microenvironment, or may become inhibited. Markers of DC differentiation/activation, such as CD83 and other molecules, may therefore be more accurate in establishing the true capacity for antigen presentation within the tumour microenvironment. Clinical evidence for this theory is provided by recent work that shows that CD83 expression correlates directly with clinical outcome (Iwamoto *et al*, 2003). The presence of a nonstatistically significant trend in the data from analysis of both (i) CD1a density and survival; and also (ii) CD1a density and NPI suggests that CD1a^+^ DC density may be important, but this is currently unclear. However, an association between CD1a expression may reach statistical significance at the 10-year time point from diagnosis, and clearly later analysis is needed to investigate this aspect. Further studies are necessary to delineate the role of DC in relation to survival in human breast cancer, and the mechanisms for enhancing *in vivo* DC function. Immunotherapeutic methods for attracting DC into, and activating DC within, the tumour microenvironment are required to improve the antitumour immune response. The long dormant periods that are clinically observed between initial diagnosis/surgery and subsequent recurrence in the clinical setting strongly indicate that local mechanisms are operational within the tumour microenvironment and are capable of successfully limiting tumour growth. Understanding DC function and activation may explain the mechanism for this phenomenon and reveal new clinically useful therapeutic tools.
